# Chemical Composition and Biological Activities of the Essential Oils from *Duguetia lanceolata* St. Hil. Barks

**DOI:** 10.3390/molecules170911056

**Published:** 2012-09-13

**Authors:** Orlando V. Sousa, Glauciemar Del-Vechio-Vieira, Maria S. Alves, Aílson A. L. Araújo, Míriam A. O. Pinto, Maria P. H. Amaral, Mírian P. Rodarte, Maria A. C. Kaplan

**Affiliations:** 1 Department of Pharmaceutical Sciences, Faculty of Pharmacy, Federal University of Juiz de Fora, São Pedro, Juiz de Fora, Minas Gerais, 36036-330, Brazil; Email: glauciemar@gmail.com (G.D.-V.-V.); alves_ms2005@yahoo.com.br (M.S.A.); ailson.luz@ufjf.edu.br (A.A.L.A.); miriamaop@yahoo.com.br (M.A.O.P.); penhaufjf@yahoo.com.br (M.P.H.A.); mirianpereira.rodarte@ufjf.edu.br (M.P.R.); 2 Department of Natural Products Research, Center of Health Sciences, Federal University of Rio de Janeiro, Ilha do Fundão, Rio de Janeiro, Rio de Janeiro, 21941-590, Brazil; Email: makaplan@uol.com.br

**Keywords:** *Duguetia lanceolata*, Annonaceae, essential oil, antimicrobial activity, brine shrimp lethality bioassay

## Abstract

Essential oils of *Duguetia lanceolata* barks, obtained at 2 (T2) and 4 h (T4), were identified by gas chromatography and gas chromatography/mass spectrometry. β-Elemene (12.7 and 14.9%), caryophyllene oxide (12.4 and 10.7%) and β-selinene (8.4 and 10.4%) were the most abundant components in T2 and T4, respectively. The essential oils inhibited the growth of *Staphylococcus aureus*, *Streptococcus pyogenes*, *Escherichia coli* and *Candida albicans*. The essential oils were cytotoxic against brine shrimp. The extraction time influenced the chemical composition and biological activities of essential oils obtained from the barks of *D. lanceolata*.

## 1. Introduction

Plant and their essential oils are potentially useful sources of bioactive compounds. Some studies have described the biological activities of essential oils against many different types of agents, including important organisms of clinical relevance [1,2]. Among the main constituents found in the essential oils, the mono- and sesquiterpenes, phenylpropanoids and benzenoid oxygenated derivatives are highlighted. These substances are responsible for the fragrance and for different biological properties [3].

The genus *Duguetia*, Annonaceae family, is composed by around 100 species and possesses as chemical markers alkaloids and acetogenins [4,5]. However, essential oils have also been identified in different species of this genus [6]. Considering the biological activities, essential oils from *Duguetia gardneriana* Mart. and *Duguetia moricandiana* Mart. showed antimicrobial activity [7], while such constituents obtained from *Duguetia glabriuscula* R. E. Fries (R.E. Fries) were active against *Artemia salina* [8].

*Duguetia lanceolata* St. Hil., popularly known as pindaíba, beribá or pinhão, is a pereninnial species distributed in several States of Brazil [5]. This species is native in this country and found in the Cerrado and Atlantic Forest, especially in Minas Gerais, Mato Grosso and São Paulo States. In folk medicine, this plant has been used as an anti-inflammatory, cicatrizing and antimicrobial agent [9]. Pharmacological studies have demonstrated that *D. lanceolata* presented antinociceptive, anti-inflammatory and antiplasmodial activities [9,10,11].

Considering that we did not find any previously published original articles about essential oils of *D. lanceolata* and in order to provide the scientific basis for the medicinal use of this species, the present study was designed to evaluate the chemical composition and the biological activities of essential oils from barks of this plant. The influence of the extraction time in the obtainment of essential oils in these two propositions for investigation was also analyzed.

## 2. Results and Discussion

This is the first time that the chemical composition of *D. lanceolata* is described in the literature. According to the Table 1, seventy-two compounds were identified in the essential oils from *D. lanceolata* barks, classified as terpenes (mono- and sesquiterpenes) and hydrocarbons. This total number of detected components (72) was found in the first two hours (T2) and fifty-one in the other four hours (T4). Among those identified in T2, twenty-five were monoterpenes and forty-seven were sesquiterpenes. Considering the time T4, nine were monoterpenes and forty-two were sesquiterpenes. The relation between the monoterpenes and the extraction time was T2 = 2.8 × T4, while for the sesquiterpenes was T2 = 1.1 × T4, demonstrating a relevant loss of monoterpenes.

*trans*-Pinocarveol (1.4 and 0.4%) and myrtenol (0.8 and 0.5%) were the most abundant monoterpenes observed in both times T2 and T4, with reduction of 71.4% and 37.5%, respectively (Table 1). Considering the twenty-five monoterpenes identified in T2, sixteen were absent in T4. Probably, the volatility and the time of hydrodistillation (4 h) caused a greater loss of this chemical class of secondary metabolite. Thus, these factors could influence the biological activities.

Sesquiterpenoids represented the highest number of constituents detected in the essential oil of *D. lanceolata* barks*.* β-Elemene (12.7 and 14.9%), caryophyllene oxide (12.4 and 10.7%), β-selinene (8.4 and 10.4%), β-eudesmol (6.8 and 7.9%), humulene epoxide II (7.4 and 6.8%), ledol (3.9 and 3.9%), viridiflorol (3.5 and 3.0%), khusinol (3.6 and 5.0%), β-senensal (3.2 and 5.4) and patchouli alcohol (3.2 and 3.8%) were the most concentrated components in T2 and T4, respectively (Table 1). It is possible that these constituents can represent important chemical markers to the essential oil of this species.

**Table 1:** Constituents of the essential oils from *D. lanceolata* barks.

Compound	RI ^a^	Concentration (%)		Identification
		2 h	4 h	
α-Pinene	937	0.1	-	RI, GC-MS
Verbenene	984	0.1	-	RI, GC-MS
β-Pinene	1018	0.1	-	RI, GC-MS
*p*-Cymene	1075	0.7	-	RI, GC-MS
Limonene	1081	0.4	-	RI, GC-MS
*p*-Cymenene	1153	0.2	0.1	RI, GC-MS
Dehydrosabina ketone	1194	0.1	-	RI, GC-MS
α-Campholenal	1202	0.1	-	RI, GC-MS
*trans*-Pinocarveol	1215	1.4	0.4	RI, GC-MS
*cis*-Verbenol	1221	0.4	-	RI, GC-MS
*trans*-*p*-Menth-2-en-1-ol	1225	0.1	-	RI, GC-MS
Camphor	1235	0.1	-	RI, GC-MS
Menthofuran	1240	0.1	-	RI, GC-MS
*p*-Mentha-1,5-dien-8-ol	1244	0.2	0.2	RI, GC-MS
Umbellulone	1251	0.1	-	RI, GC-MS
Terpin-4-ol	1256	0.4	0.1	RI, GC-MS
*p*-Cymen-8-ol	1265	0.2	-	RI, GC-MS
α-Terpineol	1270	0.1	0.1	RI, GC-MS
Myrtenol	1278	0.8	0.5	RI, GC-MS
Verbenone	1292	0.3	0.1	RI, GC-MS
*trans*-Carveol	1302	0.2	0.1	RI, GC-MS
Citronellol	1317	0.4	-	RI, GC-MS
Cumin aldehyde	1323	0.1	-	RI, GC-MS
Carvone	1328	0.1	-	RI, GC-MS
Bornyl acetate	1374	0.7	0.4	RI, GC-MS
α-Cubebene	1440	0.1	0.1	RI, GC-MS
Cyclosativene	1457	1.4	1.4	RI, GC-MS
α-Copaene	1466	0.3	0.3	RI, GC-MS
β-Elemene	1491	12.7	14.9	RI, GC-MS
( *Z*)-Caryophyllene	1497	0.2	0.1	RI, GC-MS
α-Gurjunene	1502	0.2	-	RI, GC-MS
( *allo*)-Aromadendrene	1531	0.1	0.1	RI, GC-MS
β-Chamigrene	1539	0.1	0.1	RI, GC-MS
γ-Muurolene	1553	0.3	-	RI, GC-MS
γ-Curcumene	1571	1.1	1.1	RI, GC-MS
β-Selinene	1587	8.4	10.4	RI, GC-MS
Valencene	1589	0.3	0.3	RI, GC-MS
α-Selinene	1592	0.6	0.6	RI, GC-MS
Cuparene	1596	0.4	0.4	RI, GC-MS
Germacrene A	1604	0.8	0.6	RI, GC-MS
α-Bulnesene	1608	0.6	0.6	RI, GC-MS
γ-Cadinene	1611	0.5	0.5	RI, GC-MS
δ-Cadinene	1623	1.2	1.4	RI, GC-MS
( *Z* )-Nerolidol	1630	1.2	0.8	RI, GC-MS
8,14-Cedranoxide	1639	0.5	0.3	RI, GC-MS
α-Calacorene	1642	0.9	1.2	RI, GC-MS
Occidentalol	1651	1.4	1.3	RI, GC-MS
Elemol	1655	0.3	-	RI, GC-MS
Ledol	1667	3.9	3.9	RI, GC-MS
Longipinanol	1670	2.4	0.3	RI, GC-MS
Caryophyllene oxide	1690	12.4	10.7	RI, GC-MS
Gleenol	1697	0.4	-	RI, GC-MS
Davanone	1700	0.5	1.0	RI, GC-MS
Viridiflorol	1707	3.5	3.0	RI, GC-MS
Humulene epoxide II	1716	7.4	6.8	RI, GC-MS
Hinesol	1725	0.2	0.8	RI, GC-MS
*Epi*-α-Cadinol	1733	3.2	3.0	RI, GC-MS
Cubenol	1735	0.6	-	RI, GC-MS
α-Muurolol	1747	1.0	0.9	RI, GC-MS
β-Eudesmol	1759	6.8	7.9	RI, GC-MS
Patchouli alcohol	1763	3.2	3.8	RI, GC-MS
Lyral	1773	1.2	1.2	RI, GC-MS
Cadalene	1780	1.7	2.2	RI, GC-MS
Khusinol	1788	3.6	5.0	RI, GC-MS
( *E*)-Asarone	1810	0.8	1.1	RI, GC-MS
*cis*-14-Muurolol-5-en-4-one	1817	0.5	0.8	RI, GC-MS
Germacrone	1833	0.8	0.5	RI, GC-MS
β-Sinensal	1862	3.2	5.4	RI, GC-MS
Caryophyllene acetate	1867	0.8	2.0	RI, GC-MS
n-Heptadecane	1915	0.2	0.2	RI, GC-MS
*n*-Nonadecane	2022	0.1	0.1	RI, GC-MS
*n* -Eicosane	2129	0.1	0.2	RI, GC-MS
***Total***	-	99.6	99.3	-
***Monoterpene hydrocarbons***	-	1.6	0.1	-
***Oxygenated monoterpenes***	-	5.9	1.8	-
***Sesquiterpene hydrocarbons***	-	31.9	36.3	-
***Oxygenated sesquiterpenes***	-	59.8	60.5	-
***Hydrocarbons***	-	0.4	0.5	-
***Unidentified***	-	0.4	0.7	-
***Yield (%)***	-	0.5	0.5	-

^a^ Retention index on HP-5 MS column.

The increased contents of some sesquiterpenes in T4 can be due to the loss of monoterpenes during the extraction procedure. It was also observed that the constituents with the highest content in T2 remained high in T4. Thus, the concentrations of constituents identified were 99.6 and 99.3% in T2 and T4, respectively (Table 1).

The absence of five sesquiterpenes in T4 may be attributed to the low concentration of these constituents in T2, which ranged from 0.2% to 0.6% and to the quality of the oil extracted from barks of different parts of the stem. Therefore, the influence of the time of hydrodistillation, as described with the monoterpenes, is an important factor to be observed during the extraction of essential oils. Moreover, the water, acidity and temperature involved in the hydrodistillation procedure may also cause hydrolysis, rearrangements, isomerizations, racemizations and oxidations, altering the molecular structures [12,13].

Although differences are visualized in the chemical composition of essential oils of the genus *Duguetia*, some constituents are conserved in several species. α-Pinene, β-pinene, *p*-cymene, limonene, γ-muurolene, valencene, γ-cadinene and δ-cadinene found in *D. lanceolata* have also been observed in *D. asterotricha* [14], while (*allo*)-aromadendrene, δ-cadinene and viridiflorol were identified in *D. glabriuscula* [8]. The main compounds found in *D. eximia* were α-eudesmol and spathulenol [6]. Considering an example of the difference observed in the parts of the same species, the leaf and fine stems oil of *D. flagellaris* was dominated by spathulenol and α-muurolol, while the bark oil was mainly constituted by germacrene D, cyperene, α-muurolol, humulene epoxide II and spathulenol [6]. The major components identified in the oil of *D. pycnastera* were spathulenol, *allo*-aromadendrene, germacrene D and elemol. The oil of *D. riparia* contained spathulenol, caryophyllene oxide and α-pinene as their main compounds [6]. The oil of *D. trunciflora* was dominated by α-pinene, bicyclogermacrene, bulnesol, spathulenol, guaiol, globulol and humulene epoxide II; the bark oil was dominated by β-phellandrene, guaiol and α-cadinol [6]. Germacrene D, viridiflorene, β-pinene, α-pinene and β-caryophyllene were found to be the major individual constituents of *D. gardneriana* oil [7]. The leaf oil of *D. moricandiana* was dominated by germacrene D, α-pinene, viridiflorene, β-pinene and β-caryophyllene [7]. Possibly, the conservation of these constituents in species of the genus *Duguetia* may have chemotaxonomic significance to maintain similar morphologic and biochemistry characteristics, which will determine the biosynthesis of their secondary metabolism.

Considering the biological properties, the antimicrobial activity of the essential oils from barks of *D. lanceolata* was tested against representative microbial groups using ATCC reference strains. Using disks impregnated with 5, 10 and 25 mg of essential oils obtained in the times T2 and T4, an inhibition zone of the bacteria growth was observed with Gram-positive *Staphylococcus aureus* and *Streptococcus pyogenes* and Gram-negative *E. coli*, but not with *Pseudomonas aeruginosa* (Table 2). The essential oil obtained in the time T2 was more active against the fungi *C. albicans*. Despite being active against Gram-positive bacteria, the activity of the essential oils demonstrated against *S. aureus* was smaller when compared as chloramphenicol used as an antibiotic control. The MIC data confirmed the activity against the tested microorganisms, as shown in Table 3. The MIC values ranged from 20 to 125 µg/mL and 2 to 8 µg/mL for oils and chloramphenicol, respectively, and was 15 µg/mL for nystatin (Table 3).

**Table 2:** Antimicrobial activity of the essential oils from *Duguetia lanceolata* barks.

Microorganism	Inhibition zone (mm)						
	Essential oil T2 (mg)			Essential oil T4 (mg)			Control
	5	10	25	5	10	25	
*Staphylococcus aureus*	10.5	10.5	15.0	9.5	10.0	12.5	26.0
*Streptococcus pyogenes*	11.0	12.5	18.0	10.0	11.0	14.0	28.0
*Escherichia coli*	-	-	10.0	-	-	-	25.0
*Pseudomonas aeruginosa*	-	-	-	-	-	-	21.0
*Candida albicans*	-	10	14	-	-	12	22.0

Experiments were done in duplicate and results were mean values.

**Table 3:** Minimal inhibitory concentration (MIC) of the essential oils from *Duguetia lanceolata* barks.

Microorganism	MIC (μg/mL)		
	Essential oil T2	Essential oil T4	Control
*Staphylococcus aureus*	60	125	2
*Streptococcus pyogenes*	20	40	4
*Escherichia coli*	-	-	8
*Pseudomonas aeruginosa*	-	-	-
*Candida albicans*	60	100	15

Experiments were done in duplicate and results were mean values.

Few works on the antibacterial activity of essential oils extracted from plants of the genus *Duguetia* are found in literature. One of these studies showed that the essential oil of *D. gardneriana* had activity against *S. aureus* and *Candida guilliermondii*, while the essential oil of *D. moricandiana* was more active against *S. aureus* and *Candida albicans* [7]. Several constituents of the oils of these species are also present in the essential oils from barks of *D. lanceolata.* It is possible that the antimicrobial activity demonstrated by the oil extracted from *D. lanceolata* could be attributed to these components.

It has been suggested that the antimicrobial activity of essential oils is attributable to the presence of compounds such as alcohols, aldehydes, alkenes, esters and ethers [15], some found in the oil of *D. lanceolata*. For instance, the essential oil of *D. lanceolata* contains substances as δ-cadinene, α-terpineol and elemol, found in several vegetal species, which have demonstrated bacteriostatic and antiseptic activities [16]. *p*-Cymene, β-pinene, humulene epoxide II and caryophyllene oxide identified in *Solanum erianthum* and *Solanum macranthum* also have inhibited the growth of microorganisms [17]. In addition, caryophyllene oxide, α-terpineol and limonene displayed antifungal activity against onychomycosis, *Candida albicans* and *Cryptococcus neoformans* [18,19]. However, independent on the involved species [20], it is important to mention that the antimicrobial activity could be due the synergistic action of different compounds observed in this plant.

The essential oils from barks of *D. lanceolata* obtained in the times T2 and T4 were poisonous against *Artemia salina* with following LC_50_ values: 49.0 (30.2–79.2) μg/mL and 60.7 (37.1–99.3) μg/mL, respectively (Table 4). According to the data presented in the Table 4, the results showed that the essential oil T2 was around 9 times and essential oil T4 was around seven times more poisonous than thymol (LC_50_ = 457.9 μg/mL), the reference substance.

**Table 4:** Toxicity of the essential oils from *Duguetia lanceolata* barks.

Tested product	LC_50_ (μg/mL)	Confidence interval (95%)
Essential oil T2	49.0	30.2–79.2
Essential oil T4	60.7	37.1–99.3
Thymol ^a^	457.9	318.6–658.1

^a^ Reference drug.

Species of the genus *Duguetia* have been studied by using the *A. salina* bioassay, a simple model applied to the toxicity investigation [21]. For example, the essential oil from leaves of *D. glabriuscula* (*allo*-aromadendrene and bicyclogermacrene—main constituents) presented a potent toxicity on *A. salina* with LC_50_ 1.6 μg/mL [8]. Despite the concentration of 0.1% in oils of *D. lanceolata*, *allo*-aromadendrene can contribute with the toxicity in this assay because this compound produced LC_50_ 7.8 μg/mL, demonstrating to be one of the active substances responsible for this event [8]. In addition, hexanic and methanolic extracts of *D. glabriuscula* were toxic against *A. salina* [8].

## 3. Experimental

### 3.1. Plant Material and Preparation Procedure

*Duguetia lanceolata* St. Hil. was collected in the city of Juiz de Fora, Minas Gerais State, Southeast region of Brazil. A voucher specimen identified by Dr. Fátima Regina Gonçalves Salimena was deposited in the Herbarium of the Departamento de Botânica of the Universidade Federal de Juiz de Fora (CESJ number 29750). The barks were removed from the discarded branches after pruning, submitted to drying at room temperature and triturated for the extraction of the essential oil.

### 3.2. Extraction of the Essential Oils

Essential oils were obtained from 100 g *D. lanceolata* barks with 5% of humidity and 7% of total ashes by hydrodistillation using a Clevenger apparatus for 2 h (T2) and 4 h (T4) at 100 °C. After, the essential oils were dried with anhydrous sodium sulphate and stored under refrigeration at −18 °C for analysis (the yield was 0.5% v/w).

### 3.3. Gas Chromatographic Analysis

Capillary gas chromatography was performed using a Hewlett-Packard 6890 gas chromatograph under the following conditions: fused silica capillary column HP-5 (5% diphenyl and 95% dimethylpolysyloxane, 60 m × 0.25 mm, 0.25 μm film thickness); helium as carrier gas (1 mL/min); and temperature programming from 70 to 290 °C (2 °C/min); injector temperature 270 °C and detector temperature 300 °C.

### 3.4. Gas Chromatography/Mass Spectrometry Analysis (GC/MS)

The GC/MS analysis of the essential oils was performed on a Hewlett Packard series 6890 gas chromatograph coupled to HP5972 mass spectrometer under the following analytical conditions: ZB-5MS column (30 m × 0.25 mm × 0.25 μm film thickness); helium (1 mL/min); programmed temperature 60−240 °C (3 °C/min); injector temperature (260 °C) and interface (200 °C); ionization energy, 70 eV; scan range, 30–300 amu; scan time, 1 s. Compound identification was based on the comparison of retention index (determined relatively to the retention times of a *n*-alkanes series), mass spectra and the NIST spectrometer data bank, as well as comparison with literature data [22,23].

### 3.5. Representative Microbial Groups by ATCC Reference Strains

The essential oils of *D. lanceolata* were tested against a panel of representative microorganisms including *Staphylococcus aureus* (ATCC 6538), *Streptococcus pyogenes* (ATCC 19615), *Escherichia coli* (ATCC 8739), *Pseudomonas aeruginosa* (ATCC 15442) and *Candida albicans* (ATCC 10231) which belong to the American Type Culture Collection (ATCC).

### 3.6. Screening for Antimicrobial Activity

The antimicrobial activity was tested through agar diffusion method [24]. Mueller Hinton Agar was used as the standard test medium for bacteria, with exception of *Streptococcus pyogenes* (in this case, the Mueller Hinton Agar was supplemented with Sheep Blood at 8%), and Sabouraud Agar for yeast. Overnight broth cultures were prepared, adjusted in peptone-physiological salt solution (1 g peptone and 8.5 g/L NaCl) to yield approximately 10^6^ bacteria/mL and 10^5^ conidia/mL. The agar plates were prepared in 90 mm Petri dishes with 22 mL of agar medium giving a final depth of 4 mm. Sterile cellulose discs, diameter 6 mm, were placed on the inoculated agar surfaces with 100 μL of diluted oil in hexane. Each 100 μL had 5, 10 or 25 mg of essential oil. Hexane (100 μL) was used as negative control. All plates were aerobically incubated at 35 ± 2 °C for 18–24 h (bacteria) and at 22 °C for 48 h (yeast). The antimicrobial activity was estimated by measuring the radius of the inhibition zone (mm). Each test was performed in duplicate and the results were shown as means. 20 μL of chloramphenicol (1 mg/mL) for bacteria and 20 μL of nystatin (1 mg/mL) for the yeast were used as positive controls.

### 3.7. Determination of the Minimum Inhibitory Concentration

The broth microdilution method recommended by the Clinical and Laboratory Standards Institute was used to determine the minimum inhibitory concentration (MIC) [25,26]. Antibacterial activity test was performed in Mueller-Hinton broth and for antifungal test RPMI-1640 medium with L-glutamine, buffered with MOPS buffer was used. The inoculum densities were approximately 5 × 10^5^ CFU/mL and 0.5–2.5 × 10^3^ CFU/mL for bacteria and fungi, respectively. Each essential oil was dissolved in Tween 80 and sterile distilled water. Final two fold concentrations were prepared in the wells of the microtiter plates, between 2,000–10 μg/mL. Chloramphenicol and nystatin were used as reference antibiotics for bacteria and fungi, respectively (64–0.0625 μg/mL). Microtiter plates were incubated at 35 ± 2 °C for 18–24 h for bacteria and at 22 °C for 48 h for yeast. After the incubation period, MIC values were defined as the lowest concentration of the oils that inhibits the visible growth of microorganisms.

### 3.8. Brine Shrimp Lethality Bioassay

The artificial seawater used in the experiments presented the following composition: NaCl 24 g/L, CaCl_2_·2H_2_O 1.5 g/L, KBr 0.1 g/L, KCl 0.7 g/L, Na_2_SO_4_ 4.0 g/L, NaHCO_3_ 0.3 g/L, MgCl_2_·6H_2_O 11 g/L. The essential oils were dissolved in tween 80 and DMSO (1:1) followed by artificial seawater. Ten shrimps (*Artemia salina* Leach) were transferred into test tubes in quadruplicate, containing the following essential oil concentrations: 10, 50, 100, 500 and 1,000 μg/mL. The tubes were maintained under illumination. Survivors were counted 24 h after exhibition to the oil. Thymol was used as standard [21]. CL_50_’s and 95% confidence intervals were from the 24-hour counts using the probit analysis method [27].

## 4. Conclusions

Based on our results we can conclude that β-elemene, caryophyllene oxide and β-selinene were the main constituents of the essential oils from *Duguetia lanceolata*. Both oils (T2 and T4) demonstrated antimicrobial activity against *Staphylococcus aureus*, *Streptococcus pyogenes* and *Candida albicans* reference strains tested and were poisonous against *Artemia salina*. However, even a small variation in the total chemical composition of essential oils may contribute for different levels of biological activities expression.
